# Nurses' adverse event reporting attitudes and related factors: a cross-sectional study in maternal and child specialized hospitals in China

**DOI:** 10.3389/fpubh.2024.1434387

**Published:** 2024-12-06

**Authors:** Fang Wu, Xin Wang, Shaochuan Chen, Huimin Li, Huiqiong Xie

**Affiliations:** Nursing Department, Chendu Women's And Children's Central Hospital, School of Medicine, University of Electronic Science and Technology of China, Chengdu, Sichuan, China

**Keywords:** maternal and child specialized hospital, nurse, adverse event, reporting attitude, related factors

## Abstract

**Objective:**

To investigate the current situation of nurses' attitude toward adverse event reporting and identify its related factors in maternal and child specialized hospitals.

**Methods:**

A questionnaire survey was conducted among 943 nurses in 18 second-level and above maternal and child specialized hospitals in Sichuan province in China. The questionnaire included general information and the Chinese version of Reporting of Clinical Adverse Effects Scale (C-RoCAES).

**Results:**

The total score of adverse events reported by nurses was 63.98 ± 8.77. The scores of the dimensions from high to low were reporting standard (3.13 ± 0.46), reporting impact (2.80 ± 0.54), reporting purpose (1.98 ± 0.66), and reporting environment (1.98 ± 0.42). Educational background (β = −1.87, *p* < 0.001), professional title (β = −3.51, *p* < 0.001), and adverse event experience (β = −7.05, *p* < 0.001) were the positively associated with higher levels of nurses' attitude toward adverse event reporting in maternal and child specialized hospitals (*p* < 0.05).

**Conclusion:**

The attitude of nurses in maternal and child specialized hospitals to report adverse events is at the middle level. Hospital managers should improve the reporting standards for adverse events, improve the hospital safety culture, strengthen the relevant training for nurses with low education and low professional titles, so as to improve nurses' awareness of adverse event reporting and reporting rate.

## 1 Introduction

Patient safety is: “A framework of organized activities that creates cultures, processes, procedures, behaviors, technologies and environments in health care that consistently and sustainably lower risks, reduce the occurrence of avoidable harm, make errors less likely and reduce the impact of harm when it does occur” (1). Ensuring the safety of patients is an important part of the delivery of health services. Developing a culture of safety is cardinal to any sustainable efforts toward patient safety improvement. Leadership commitment, transparency, open and respectful communication, learning from errors and best practices, and a judicious balance between a no blame policy and accountability are indispensable components of safety culture. A strong safety culture is not only core to reducing patient harm, it is also critical for providing a safe working environment for health workers (2).

According to the World Health Organization (WHO), around one in every 10 patients is harmed in health care and more than three million deaths occur annually due to unsafe care. In low-to-middle income countries, as many as four in 100 people die from unsafe care (3). Above 50% of harm is preventable (4). In high-income countries, up to 15% of hospital expenditure can be attributed to wastage due to safety failures. For example, the National Health Service in England paid £1.63 billion in litigation costs because of safety lapses (5). Some estimates suggest that as many as four in 10 patients are harmed in primary and ambulatory settings, while up to 80% of this harm can be avoided (6). Patient harm potentially reduces global economic growth by 0.7% a year. On a global scale, the indirect cost of harm amounts to trillions of US dollars each year (7). Common adverse events that may result in avoidable patient harm are medication errors, unsafe surgical procedures, health care associated infections, diagnostic errors, patient falls, pressure ulcers, patient misidentification, unsafe blood transfusion and venous thromboembolism (8).

Adverse events refer to unsafe or adverse events that are actively discovered by staff in the hospital and are caused by various factors other than the natural course of the patient's own disease, including four categories: hidden events, non-consequence events, consequence events, and warning events (9). Adverse events not only prolong hospital stays, increase healthcare costs, and may even lead to physical dysfunction, but also have a negative psychological impact on the patients concerned (10). In one meta-analysis, preventable adverse events occurred in 6%, with 12% of preventable adverse events being serious or resulting in death (11). According to the 2018 China National Medical Service and Quality and Safety Report, the incidence of adverse events in medical institutions in China is only 0.61% (12). There is a concerning problem that the low number of reported adverse events in statistics may be related to the lack of notification of these events. When adverse events go unreported, it becomes difficult to assess the full extent of potential harm and take appropriate measures to address and prevent similar incidents in the future. Additionally, a lack of notification may result in a false sense of security, as it gives the impression that there are fewer risks than there actually are. It is essential to encourage and improve the reporting mechanisms for adverse events to ensure a more accurate picture of their occurrence and to safeguard public health and safety. The majority of patient safety incidents involve nurses, and more than half of them occur in patient rooms. More than 50% of nurses have experienced adverse events (13).

The reporting and management of adverse events in the nursing process is an important measure to ensure the quality of nursing. Escalation management of adverse events can help medical professionals understand and learn from the different errors that occur around them, help identify problems in the system, and improve the system to improve patient safety (14). Despite the efforts of the government and hospitals to improve the reporting rate of adverse events, the number of recorded adverse events is still significantly lower than the actual incidence rate. How to improve the rate of adverse event reporting is an urgent problem to be solved, and the attitude of adverse event reporting is an important predictor of adverse event reporting (15).

Pregnant women and children belong to a special group of patients, and the nursing workload associated with them is large, the degree of risk is high, and the professionalism is strong (16, 17), especially for children, who have weak self-protection ability, lack of expression ability, low degree of cooperation, and are very prone to adverse events. Falls, infusion-related adverse events, and dosing errors have been reported in the literature to be the most common adverse events in maternal and child hospitals (18, 19). Therefore, in order to understand the current situation and influencing factors of nursing staff's willingness to report adverse events in maternal and child specialized hospitals, this study investigated and analyzed the nurses of 18 secondary and above maternal and child specialized hospitals in Chengdu, and the results are as follows.

## 2 Methods

### 2.1 Study design

This study used a cross-sectional design and employed an online questionnaire to survey.

### 2.2 Participants

Nurses from 18 maternal and child hospitals who met the inclusion and exclusion conditions were selected as the research subjects, and the whole cluster was included in the questionnaire survey after informed consent.

Inclusion criteria: nurses who work in second-level and above maternal and child specialized hospitals; nurses on duty during the survey;registered nurse; informed consent to participate in this study.

Exclusion criteria: nurses who are on leave or out to study during the survey; nurses in non-clinical departments, nurses who come to the hospital for advanced training and nurses who are trained in a standardized manner.

### 2.3 Research tools

#### 2.3.1 General information questionnaire

It includes the age, sex, education level, professional title, department, hospital properties, position, employment status, work experience, adverse event experience, adverse event reporting experience, and whether you know the reporting process of adverse events.

#### 2.3.2 Reporting of clinical adverse effects scale

The Reporting of Clinical Adverse Effects Scale (RoCAES) developed by Wilson et al. (14) in 2003 and translated by Zhou et al. (20) in China to assess the attitude of medical staff toward adverse event reporting. The Chinese version of RoCAES uses the Likert scale (1–4) (1 = strongly agree, 4 = strongly disagree), a total of 4fourdimensions (reporting standard, reporting impact, reporting purpose, and reporting environment), 25 items, the higher the score, the lower the willingness to report adverse events. C-RoCAES Cronbac α coefficient was 0.966, the half-component reliability of the four dimensions was 0.949, 0.871, 0.795, 0.537, and the test-retest reliability was 0.794, 0.851, 0.735, and 0.657. The results indicated that the C-RoCAES had good reliability and validity, and could be used to evaluate nurses' adverse event reporting attitudes.

### 2.4 Ethical considerations

This survey is completely anonymous and voluntary. The first page of the questionnaire is an informed consent form, which explains the purpose of the study, the potential risks and benefits to nurses, the time it takes to fill out the questionnaire. Participants can withdraw from the study at any time without any impact on them. The data is only used for scientific research and can only be accessed by researchers using a password. This study has been approved by the Ethics Committee of Chengdu Women's and Children's Central Hospital [Research Ethics 2022 (27)]. The study follows the Declaration of Helsinki of the World Medical Association.

### 2.5 Data collection

Questionnaires were used to conduct an online survey. After the questionnaire was collected, the members of the research team checked the answers, and excluded the invalid questionnaires with an answer time outside the 95% range. The recovery rate was 99.7%.

### 2.6 Data analysis

SPSS 26.0 was used for data analysis. The counts were described by frequency and composition ratio, and the mean ± standard deviation was used to describe the continuous data, and independent samples *t*-test, analysis of variance, correlation analysis and regression analysis were used, and the test level was α = 0.05.

## 3 Results

### 3.1 General information

The participants of this study were 943 nurses, of which 78.5% were nurses in tertiary hospitals and 21.5% were nurses in secondary hospitals, mainly aged 26–35 years old, 99.3% were females, and most of them had bachelor's degree or above, most of whom were junior titles. Among the 943 nurses, 79.7% were clinical nurses, 95.8% of the nurses were clearly aware of the adverse event reporting process, 56.5% of the nurses had experienced adverse events, and 56.6% of the nurses had reported adverse events, see Table 1 for details.

**Table 1:** General information (*n* = 943).

**Variables**		**Sex**	***n***	**Percentage (%)**
Age (years)	≤ 25	Women	134	14.2
		Men	0	0
	26–30	Women	312	33.1
		Men	3	0.3
	31–35	Women	271	28.8
		Men	3	0.3
	36–40	Women	100	10.6
		Men	0	0
	≥41	Women	119	12.6
		Men	1	0.1
Education level	Secondary technical	Women	10	1.1
		Men	0	0
	Associate degree	Women	314	33.3
		Men	1	0.1
	Bachelor degree	Women	607	64.4
		Men	6	0.6
	Master degree or above	Women	5	0.5
		Men	0	0
Professional title	Junior	Women	638	67.7
		Men	6	0.6
	Intermediate	Women	249	26.4
		Men	1	0.1
	Senior	Women	49	5.2
		Men	0	0
Department	Gynecology	Women	87	9.2
		Men	0	0
	Obstetrics	Women	228	24.2
		Men	0	0
	Pediatrics	Women	202	21.4
		Men	0	0
	Operating room	Women	58	6.2
		Men	1	0.1
	Outpatient	Women	112	11.9
		Men	2	0.2
	Emergency	Women	56	5.9
		Men	1	0.1
	Intensive Care Unit	Women	22	2.4
		Men	3	0.3
	Others	Women	171	18.1
		Men	0	0
Hospital properties	Public tertiary hospitals	Women	734	77.9
		Men	6	0.6
	Public secondary hospitals	Women	202	21.4
		Men	1	0.1
Position	Clinical front-line nurses	Women	745	79.0
		Men	7	0.7
	Nursing team leader	Women	96	10.2
		Men	0	0
	Head nurse	Women	85	9.0
		Men	0	0
	Director of nursing	Women	10	1.1
		Men	0	0
Employment status	Contractual	Women	768	81.5
		Men	6	0.6
	Permanent	Women	168	17.8
		Men	1	0.1
Work experience (years)	< 1	Women	60	6.4
		Men	2	0.2
	1–5	Women	308	32.7
		Men	1	0.1
	6–10	Women	267	28.3
		Men	2	0.2
	11–20	Women	213	22.6
		Men	2	0.2
	≥21	Women	88	9.3
		Men	0	0
Adverse event experience	Yes	Women	529	56.1
		Men	4	0.4
	No	Women	407	43.2
		Men	3	0.3
Be aware of the adverse event reporting process	Yes	Women	903	95.8
		Men	0	0
	No	Women	33	3.5
		Men	7	0.7
Adverse event reported experience	Yes	Women	528	56.0
		Men	6	0.6
	No	Women	408	43.3
		Men	1	0.1

### 3.2 The score of nurses' attitude toward reporting adverse events

The total score of adverse events reported by nurses was 63.98 ± 8.77. The scores of the dimensions from high to low were reporting standard (3.13 ± 0.46), reporting impact (2.80 ± 0.54), reporting purpose (1.98 ± 0.66), and reporting environment (1.98 ± 0.42), details shown in Table 2.

**Table 2:** Nurses' attitude score toward reporting adverse events in maternity and child specialized hospitals.

**Questionnaire RoCAES**	**The number of item**	**Minimum score**	**Max score**	**Dimension equalization ($\bar{\text{x}}\;\pm \;\text{s}$)**	**Total score ($\bar{\text{x}}\;\pm \;\text{s}$)**
Reporting standard	9	16	36	3.13 ± 0.46	28.18 ± 4.13
Reporting environment	8	8	28	1.98 ± 0.42	15.87 ± 3.39
Reporting impact	5	5	20	2.80 ± 0.54	13.99 ± 2.71
Reporting purpose	3	3	12	1.98 ± 0.66	5.94 ± 1.99
Total	25	34	92	2.47 ± 0.37	63.98 ± 8.77

Multiple linear stepwise regression analysis was carried out with the total score of nurses' attitude toward adverse event reporting in maternal and child specialized hospitals as dependent variables, and the age, gender, educational background, professional title, department, position, hospital nature, working experience, whether they have experienced adverse events, whether they have reported adverse events, and whether they know the adverse event reporting process were used as independent variables, and the variable assignment is detailed in Table 3. The results (Table 4) showed that educational background, professional title, and whether or not they had experienced adverse events were the influencing factors of nurses' attitude toward reporting adverse events.

**Table 3:** Variable assignment.

**Variables**	**Sex**	**Variable assignment**	**Variables**	**Sex**	**Variable assignment**
Age			Hospital properties		
≤ 25	Women	0	Public tertiary hospitals	Women	0
	Men	0		Men	0
26–30	Women	1	Public secondary hospitals	Women	1
	Men	1		Men	1
31–35	Women	2	Position		
	Men	2	Clinical front-line nurses	Women	0
36–40	Women	3		Men	0
	Men	3	Nursing team leader	Women	1
≥41	Women	4		Men	1
	Men	4	Head nurse	Women	2
Education level				Men	2
Secondary technical	Women	0	Director of nursing	Women	3
	Men	0		Men	3
Associate degree	Women	1	Work experience		
	Men	1	< 1	Women	0
Bachelor degree	Women	2		Men	0
	Men	2	1–5	Women	1
Master degree or above	Women	3		Men	1
	Men	3	6–10	Women	2
Professional title				Men	2
Nurse	Women	0	11–20	Women	3
	Men	0		Men	3
Nurse Practitioner	Women	1	≥21	Women	4
	Men	1		Men	4
Nurse in charge	Women	2	Adverse event experience		
	Men	2	Yes	Women	1
Deputy chief nurse and above	Women	3		Men	1
	Men	3	No	Women	0
Department				Men	0
Gynecologic	Women	0	Be aware of the adverse event reporting process		
	Men	0	Yes	Women	1
Pediatrics	Women	1		Men	1
	Men	1	No	Women	0
Operating room	Women	2		Men	0
	Men	2	Adverse event reported experience		
Outpatient and emergency	Women	3	Yes	Women	1
	Men	3		Men	1
Intensive care unit	Women	4	No	Women	0
	Men	4		Men	0
Others	Women	5			
	Men	5			

**Table 4:** Multiple linear regression analysis of nurses' adverse event reporting attitudes in maternity and child specialized hospitals.

**Independent variables**	**β**	**SE**	**β^′^**	***t***	***p***
Constant	75.53	0.85	—	88.62	0.000
Education level	−1.87	0.48	−0.11	−3.88	0.000
Professional title	−3.51	0.35	−0.28	−9.92	0.000
Adverse event experience	−7.05	0.49	−0.40	−14.46	0.000

## 4 Discussion

### 4.1 Analysis of the current situation of nurses' adverse event reporting attitudes in maternity and child specialized hospitals

The results of this study showed that the total score of nurses' adverse event reporting attitude in maternal and child specialized hospital was 63.98 ± 8.77 points, which was lower than the study (21) which survey 745 nurses and midwives working in internal medicine surgical wards in nine hospitals in a large provincial city in Poland (surgical nurses 71.10 ± 7.60); internal medicine nurses 72.04 ± 7.99; midwives 71.26 ± 7.25 and the survey of 1,162 nurses in a tertiary general hospital in Henan Province (74.17 ± 8.09) (22). According to the C-RoCAES, the higher the score, the lower the willingness to report adverse events, indicating that nurses in maternal and child specialized hospitals were at the middle level of adverse event reporting. Since 2021, the Chinese National Health Commission has taken increasing the reporting rate of adverse events as a national medical quality and safety improvement goal for two consecutive years (23), although various methods and forms have been adopted at the hospital level to encourage reporting, it has not yet achieved good results, and there is a lack of authoritative medical safety event management norms and standards at the national and hospital levels (9), which may lead to the lack of awareness of adverse events among nursing staff, so their attention and participation are not high, which affects the reporting of adverse events. On the other hand, pregnant women and children belong to a special group, and their families pay more attention to pregnant women and children, and have higher requirements for medical personnel, which prompts nursing staff to pay more attention to the safety of pregnant women and children, thereby promoting nursing staff to pay more attention to the reporting of adverse events to a certain extent.

Among the four dimensions of this study, the mean score of the reporting environment and the purpose of reporting was low, indicating that the nurses in the city's maternal and child specialized hospital had a clear understanding of the purpose of adverse event reporting and a good reporting atmosphere, which was related to the vigorous promotion of medical quality and safety management at the national level, and the Chinese National Health Commission in 2022 regarded improving the reporting rate of adverse events as one of the national medical quality and safety improvement goals (23). At the same time, the non-punitive reporting system was vigorously promoted, and the 18 maternity and child hospitals surveyed in this survey have implemented a non-punitive reporting system for adverse events. Among the four dimensions, the average score of reporting standards and reporting impact was high, indicating that nurses in maternal and child hospitals did not have a sufficient grasp of the standards for reporting adverse events, and believed that small and common adverse events did not need to be reported, and reporting adverse events would hinder the development of their careers. A non-punitive reporting environment can improve health care workers' awareness of adverse events, effectively reduce their fear of reporting, and thus increase reporting rates (24).

### 4.2 Analysis of influencing factors of nurses' adverse event reporting attitude in maternity and child specialized hospitals

#### 4.2.1 Education level

The results show that education is the related factor of adverse event reporting attitude, and the higher the education level, the more positive the reporting attitude, which is consistent with the research who pointed out that with the increase of educational qualifications, the grasp of new knowledge and relevant information increases, and the deeper the awareness of safety culture, the more positive the reporting attitude (10). Among the subjects of this study, 66% of them have a bachelor's degree or above, and with the improvement of nursing staff's academic qualifications, their awareness of adverse events is more in-depth, which is conducive to the formation of hospital safety culture. Therefore, as hospital administrators, nurses should be encouraged to carry out on-the-job education and improve their academic qualifications.

#### 4.2.2 Professional title

The results of the study show that people with higher professional titles have a more positive attitude toward reporting. This is consistent with the findings: the intention to report adverse events was highest with the title of nurse in charge and above (25). The reason for the analysis may be that on the one hand, those with higher professional titles tend to work longer in the hospital than those with junior professional titles, have an in-depth understanding of the rules and regulations in the hospital, and have a clearer understanding of the process of adverse event reporting; nurses with lower professional titles are often junior nurses, and the lack of experience in reporting adverse events, the incomplete understanding of the safety culture in the hospital, and the fear of punishment after adverse event reporting lead to low willingness to report; on the other hand, most of the nurses with senior professional titles are involved in the management of the ward, and are responsible for the nursing team leader, roles such as nurse managers usually have a higher awareness of patient safety and the concept of sharing a culture of error, and are able to respond positively to adverse events, so they are more positive in reporting (25, 26). Researchers also found that nurses with senior titles were better able to detect and manage adverse events than nurses with junior titles (27).

#### 4.2.3 Experience of adverse events

Multiple regression analysis showed that the experience of adverse event reporting had a predictive effect on adverse event reporting attitudes, which was similar to the results of Majda et al. (28). Since most of the maternity and child specialized hospitals have implemented a non-punitive reporting system, nurses will not have adverse effects on themselves after reporting adverse events, and even some hospitals have implemented an adverse event reporting reward system, so nurses have a more positive reporting attitude. Some studies (29, 30) pointed out that after the implementation of the non-punitive adverse event reporting system, nursing staff can subjectively recognize the importance of reporting, and through the analysis and improvement and prevention of reported events, the incidence of adverse events can be significantly reduced.

### 4.3 Limitations

There are potential study limitations in this study. Firstly, the majority of the participants in this study were women and only seven men, this sex difference may limit the generalizability of the results of this study and their applicability to clinical practice, and future studies should take into account sex differences. Secondly, this study only surveyed nurses in a city maternal and child specialized hospital in southwest China, which limited the generalization of the research results, and the research scope should be expanded in the future to conduct a multi-center study. Thirdly, this study is a quantitative study, mainly focusing on the data of the surface layer, and it is difficult to obtain in-depth information and causes, and future research can be combined with qualitative research to investigate and analyze the reasons in depth.

## 5 Conclusion

This study concluded that the adverse event reporting attitude of nurses in maternal and child specialized hospitals was at a medium level and needed to be improved. Managers should strengthen the training of reporting standards, further implement the non-punitive reporting system, and actively create a good reporting environment. Managers should encourage clinical nurses to improve their academic qualifications and professional titles, and at the same time let nurses who have experienced adverse events report to share their knowledge of the adverse event reporting process and system, so as to promote the reporting of adverse events and ensure patient safety.

## Data Availability

The original contributions presented in the study are included in the article/Supplementary material, further inquiries can be directed to the corresponding author.
